# Albumin-Functionalized Iron Oxide Nanoparticles for Theranostics: Engineering and Long-Term In Situ Imaging

**DOI:** 10.3390/pharmaceutics14122771

**Published:** 2022-12-11

**Authors:** Anna V. Bychkova, Marina N. Yakunina, Mariia V. Lopukhova, Yevgeniy N. Degtyarev, Mikhail V. Motyakin, Vadim S. Pokrovsky, Alexander L. Kovarski, Maria G. Gorobets, Vasily M. Retivov, Derenik S. Khachatryan

**Affiliations:** 1 Emanuel Institute of Biochemical Physics of Russian Academy of Sciences, 4, Kosygina Str., Moscow 119334, Russia; 2 N.N. Blokhin National Medical Research Center of Oncology, 24, Kashirskoye Sh., Moscow 115478, Russia; 3 N.N. Semenov Federal Research Center for Chemical Physics, Russian Academy of Sciences, 4, Kosygina Str., Moscow 119991, Russia; 4 Laboratory of Experimental Oncology, Research Institute of Molecular and Cellular Medicine, RUDN University, 6, Miklukho-Maklaya Str., Moscow 117198, Russia; 5 Department of Biotechnology, Sirius University of Science and Technology, 1, Olympic Pr., Federal Territory Sirius, Krasnodarsky Kray, Sochi 354340, Russia; 6 The Federal State Unitary Enterprise, Institute of Chemical Reagents and High Purity Chemical Substances of National Research Center “Kurchatov Institute”, 3, Bogorodsky Val, Moscow 107076, Russia; 7 National Research Center “Kurchatov Institute”, 1, Akademika Kurchatova pl., Moscow 123182, Russia

**Keywords:** human serum albumin (HSA), iron oxide magnetic nanoparticles with peroxidase-like activity, free radical approach (FRA), computed tomography (CT), theranostics

## Abstract

Magnetic nanosystems (MNSs) consisting of magnetic iron oxide nanoparticles (IONPs) coated by human serum albumin (HSA), commonly used as a component of hybrid nanosystems for theranostics, were engineered and characterized. The HSA coating was obtained by means of adsorption and free radical modification of the protein molecules on the surface of IONPs exhibiting peroxidase-like activity. The generation of hydroxyl radicals in the reaction of IONPs with hydrogen peroxide was proven by the spin trap technique. The methods of dynamic light scattering (DLS) and electron magnetic resonance (EMR) were applied to confirm the stability of the coatings formed on the surface of the IONPs. The synthesized MNSs (d ~35 nm by DLS) were intraarterially administered in tumors implanted to rats in the dose range from 20 to 60 μg per animal and studied in vivo as a contrasting agent for computed tomography. The long-term (within 14 days of the experiment) presence of the MNSs in the tumor vascular bed was detected without immediate or delayed adverse reactions and significant systemic toxic effects during the observation period. The peroxidase-like activity of MNSs was proven by the colorimetric test with o-phenylenediamine (OPD) as a substrate. The potential of the synthesized MNSs to be used for theranostics, particularly, in oncology, was discussed.

## 1. Introduction

For the last decades, the applications of magnetic iron oxide nanoparticles (IONPs) for the diagnosis and treatment of various diseases have been intensively developed [1,2,3,4,5]. The main medical applications of IONPs nowadays are magnetic hyperthermia, targeted drug delivery, and the visualization of tumor tissues, preferably with magnetic resonance imaging (MRI) [6,7,8,9,10,11], but also with computed tomography (CT) [12,13,14]. In recent years, multiple areas of the medical application of magnetic iron oxide nanoparticles with enzyme-like activity (nanozymes, IONzymes) have been proposed including cancer therapy by means of ferroptosis [15,16].

Human serum albumin (HSA) is a predominant plasma protein (60% of the protein amount in plasma) often used as a component of the artificial protein coatings on the surface of nanoparticles, particularly, IONPs [17,18,19,20,21], because of its stability, moderate toxic effects, immunogenicity, high biocompatibility, and biodegradability. Albumin is the most important carrier protein in human blood for both endogenous and exogenous molecules, enabling certain drugs to remain in blood stream in quantities beyond their natural plasma solubility, decreasing their toxicity, lowering clearance rates, and increasing the circulatory half-life [22,23]. It is known that tumor cells ingest more albumin into their lysosomal compartments than normal cells [24], and albumin has a natural ability to accumulate at some disease sites [22].

In many studies devoted to the engineering of albumin-coated magnetic iron oxide nanoparticles, the coatings are mostly fixed on the surface of IONPs by physical adsorption [25,26] and/or with the addition of chemicals [27,28] and using other approaches [17]. We were the first to prove that HSA could be bound to the surface of IONPs as a result of free radical modification [29]. The free radical approach (FRA) we suggested is based on the initiation of free radical processes on the surface of IONPs [30]. The similar approach has been used to obtain nanoparticles from bovine serum albumin containing antitumor drug by hydroxyl radical oxidation via the Fenton reaction through one-step mixing [31]. In our opinion, FRA has advantages compared to the traditional methods since it does not require special chemicals or additional procedures and is based on HSA ability of free radical-trapping activity in serum [32,33,34]. The goal of the present study was to engineer the stable HSA coating on iron oxide nanoparticles using FRA and to carry out the analysis of their distribution and biological effects in rats with an inoculated hepatocellular carcinoma PC-1 cells. CT was applied to determine the distribution of magnetic nanosystems (MNSs) in tumor nodules. The side effects of MNS administration in rats were also monitored in the study.

We have proven in this study by dynamic light scattering (DLS) and electron magnetic resonance (EMR) spectroscopy that FRA allowed obtaining a stable thin HSA coating on IONPs with peroxidase-like activity. To confirm the fixation of HSA on the surface of IONPs, we used an original test based on immunoglobulin G affinity to IONPs. The concentration of MNS hydrosol taken for the CT analysis corresponded to the IONP concentration from 20 to 60 µg per tumor. The CT measurements 30 min and 14 days after their intraarterial administration showed a non-dose- and non-time-dependent contrasting of the tumor vascular bed. Therefore, the minimal dose (20 μg IONPs per tumor) appeared to be enough for the noticeable contrasting of the tumor vessels. Therefore, we expect FRA to help obtaining the best HSA coating on IONPs in terms of stability as well as a biocompatible bioinspired drug transport nanosystem based on albumin-functionalized IONPs.

## 2. Materials and Methods

### 2.1. Sample Preparation

**Figure 1:**
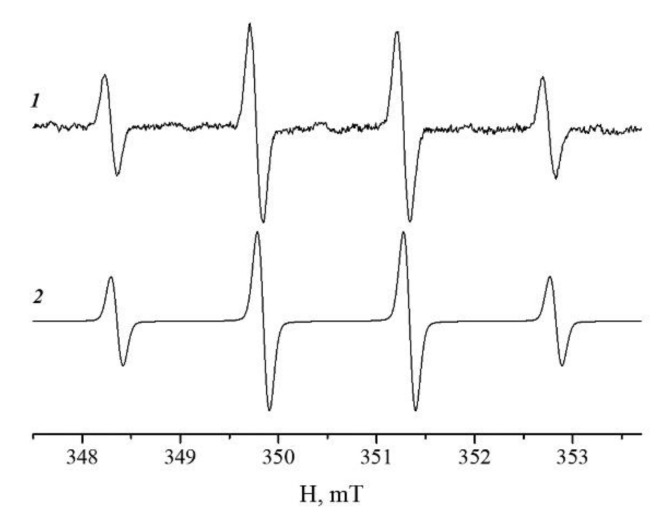
Experimental (1) and simulated (2) electron spin resonance spectra of the 5,5-dimethyl-1-pyrroline N-oxide (DMPO) spin adduct in an aqueous solution containing iron oxide nanoparticles (IONPs) and the hydrogen peroxide. The simulated spectra of the spin adducts were obtained using hyperfine coupling constants a_N_ = 1.49 mT and a_H_ = 1.50 mT from [37].

Magnetic IONPs were synthesized by the co-precipitation method and electrostatically stabilized by 0.1 M phosphate-citrate buffer (0.05 M NaCl) with pH 4.3, as presented in the previous study [35]. The IONP hydrosol (~22 mg/mL) was stored in a sealed vessel. The presence of the ferrous ions on the surface of IONPs, the catalytic/IONzyme properties of IONPs before the sample preparation as well as the free radical generation in the reaction between IONPs and hydrogen peroxide were confirmed by: (1) the formation of acrylamide gel in the mixture containing IONPs, acrylamide, N,N’-methylenebisacrylamide, and the hydrogen peroxide as described earlier [36]; (2) by the formation of 2,3-diaminophenazine in the mixture containing IONPs, o-phenylendiamine, and the hydrogen peroxide as described in Section 2.6; and (3) by the formation of the •OH adduct [37] using the spin trapping reagent 5,5-dimethyl-1-pyrroline N-oxide (DMPO) (Figure 1), as described in Section 2.4. The IONP hydrosol was 10 times diluted in 0.05 M phosphate buffer pH 6.3 and incubated at Nd-Fe-B magnets to remove the largest particles from the solution. The IONP sizes were estimated with the aid of DLS (see Section 2.3 for the measurement technique) and nanoparticle tracking analysis (NTA) (see Section S1 in the Supplementary Materials for the measurement technique and the data obtained; References [38,39] are cited in the Supplementary Materials). The buffer for the HSA coating formation was chosen according to our previous study [40].

HSA was purchased from Sigma (A1653). The aqueous HSA solution with a protein concentration of 100 mg/mL was added as 10 vol.% to the 10-fold diluted IONP hydrosol immediately under stirring on a Vortex V-1 plus (Biosan, Riga, Latvia). The C_HSA_/C_IONPs_ ratio was 10 [mg/mg]. According to the procedures we discussed elsewhere [30,36], hydrogen peroxide solution (>30%) (95321, Sigma-Aldrich, St. Louis, MO, USA) was diluted in water to be added as 3.0 vol.% to the solutions containing HSA and IONPs in a C_H2O2_/C_IONPs_ ratio [mg/mg] equal to 1/1 and incubated in a Rotator Multi Bio RS-24 (Biosan, Riga, Latvia). The sample obtained by a similar procedure with water instead of hydrogen peroxide was also created. The samples were named “NH1” (with H_2_O_2_) and “NH0” (without H_2_O_2_). The control sample “N0” consisted of IONPs only. The IONP concentration in “N0” was equivalent to the IONP concentration in “NH1” and “NH0”.

The samples were incubated overnight. Then, the magnetic separation was carried out at the magnets three times to wash out the excess components (the buffer, the protein, and the hydrogen peroxide). The data were collected for all the samples before and after magnetic separation using dynamic light scattering and electron magnetic resonance techniques to control the design of MNSs.

All samples of IONPs and HSA as well as the control samples of HSA in the presence and in the absence of H_2_O_2_ were studied by UV–Visible spectroscopy using the Bradford protein assay at each stage of their preparation including the supernatant and the precipitate samples obtained in the magnetic separation process. The protein was not detected in the supernatant obtained after the third stage of magnetic separation, which confirmed the completeness of the clean-up (removing) of excess protein. The absorption spectra were measured on a UV–Visible spectrometer SF-2000 (OKB “SPECTR”, Saint Petersburg, Russia) in quartz cells with a 1 cm optical path at a temperature of 25 °C.

All of the chemicals were of analytical grade or higher. All of the samples were prepared in double-distilled water and incubated at 25 °C.

### 2.2. The Protein Coating Stability Test

It is known [36,41,42,43] that IONPs aggregate in the presence of fibrinogen and immunoglobulin G due to the high affinity of these proteins to surface of IONPs. We hypothesized that the high-affinity proteins would substitute HSA on the IONP surface with the formation of micron-sized aggregates in the case of the low stability of the HSA coating. The effect was confirmed in [30,40]. The approach previously developed for fibrinogen (“fibrinogen test”) was used in this study for immunoglobulin G.

Human immunoglobulin G (IgG) used was purchased from the Scientific and Production Association for Immunological Preparations “Microgen” (Nizhny Novgorod, Russia) and initially treated as described earlier [36,40]. IgG was added to the solutions of the samples “NH1”, “NH0”, and “N0” before the magnetic separation to detect the HSA coating stability in the samples with different incubation times and chose the conditions to obtain the most stable coatings correspondingly. IgG was added to the solutions of the samples after magnetic separation to confirm the HSA coating stability before the injection of samples to animals. The IgG addition followed a 20-fold dilution of the samples in 0.05 M phosphate buffer pH 6.3. The final concentration of IgG in the solutions did not exceed 0.03 mg/mL. This corresponded to a C_IgG_/C_IONPs_ ratio close to 0.6 [mg/mg] and similar to that in the samples containing bare IONPs and IgG, where the formation of the aggregates took place [36].

### 2.3. Dynamic Light Scattering Measurements

The size distribution histograms of nanoparticles in the initial hydrosol, of particles in all of the samples, in the protein coating stability tests, and in the tests regarding the stability of the MNSs before their injection to animals and regarding the control of MNS sizes during the period of 3 weeks were measured three times with the aid of DLS on a Zetasizer Nano-S instrument (Malvern, UK) at a detection angle of 173° and temperature of 25 °C. Each measurement was divided into 10 runs. In accordance with the software, the runs that contained the poorest data were automatically rejected while the remaining runs were analyzed and used in the final measurement calculation.

### 2.4. Electron Magnetic Resonance Measurements

Electron magnetic resonance (EMR) spectra (the first derivative of the adsorption signal) were recorded at 25 °C using the X-band spectrometer Bruker EMX-8/2.7 (Karlsruhe, Germany). The samples were placed into the resonator of the spectrometer using glass capillaries 1.0 ± 0.1 mm i.d.

To detect the formation of hydroxyl radicals, the electron paramagnetic resonance (EPR) spectroscopy method of a spin trap based on the reaction of the short-lived radical with a spin trap, leading to the formation of a stable nitroxyl radical (spin adduct), was used [44]. 5,5-Dimethylpyrroline-N-oxide (DMPO, Abcam, Cambridge, UK) was used as the spin trap. The samples were prepared in the distilled water and contained 500 mM of H_2_O_2_, 4 µg/mL of IONPs, and 66 mM of DMPO. The microwave power was less than 2 mW to avoid saturation effects; the modulation amplitude of 100 kHz did not exceed 0.08 mT.

EMR spectra of the samples “NH1”, “NH0”, “N0” were recorded at an operating frequency of 9.87 GHz in magnetic fields of 100–550 mT. The concentration of IONPs in the experiments did not exceed 0.05 mg/mL. EMR measurements were carried out with the following instrumental settings: the microwave power of 2 mW, the modulation amplitude of 0.3 mT, magnetic field resolution of 2048 points, the time constant of 40.96 ms, and sweep time of 167.77 s.The resonance field (the position of the spectrum center, H_c_ or g-factor) was determined for all the samples before and after magnetic separation before and during the protein coating stability tests. The experimental errors of the ΔH_c_ and Δg-factor were ±0.25 mT and ±0.006, respectively. The experimental spectra are presented in absorption mode. In our opinion, this mode clearly allows one to identify the changes in the samples due to the interaction of IONPs with proteins.

We used the hydrosol of IONPs synthesized and studied earlier [35,45] as the reference data for the evaluation of the IONP concentration in the samples.

Mathematical processing of the EMR spectra was carried out using the Bruker (WINEPR and SimFonia Ver. 2.00 Rev.01) software.

### 2.5. Colorimetric Test of Peroxidase-like Activity of MNSs

The free radical generation by MNSs was estimated by the method based on the measurement of the UV/Vis absorbance for 2,3-diaminophenazine (DAP) produced due to the oxidation of o-phenylendiamine (OPD) by hydrogen peroxide in the presence of IONPs [46,47]. The DAP generation was measured in the mixtures containing the analyzed samples (“NH1”, “NH0”, “N0”) with a concentration of IONPs equal to 4 µg/mL, 0.075 mM OPD, and 9.8 mM H_2_O_2_ on a UV–Visible spectrometer SPECTROstar Nano (BMG, Germany) in 96-well plates from Greiner at a temperature of 37 °C at the wavelength (λ_max_ = 418 nm). The speed of DAP formation (V_max_, M/s) was calculated by the tangent of the angle of inclination of the initial linear section of the OD change using an extinction coefficient of 13,000 M^−1^cm^−1^ [48]. Then, the relative speeds of DAP formation V_max(sample)_/V_max(“N0”)_ (%) were evaluated for the samples “NH0” and “NH1” immediately after the magnetic separation process and for the sample “NH1” on the first, fourth, and seventh days after the magnetic separation process.

### 2.6. Computed Tomography Study

To study the contrasting properties of MNSs, we used computed tomography (CT) equipped with a PHILIPS Brilliance scanner (USA). The cross-sectional images with a 2 mm slice thickness were registered with a tube voltage of 120 kV and a tube current of 200 mA. The CT imaging reconstruction was carried out using the PHILIPS Brilliance scanner software. The degree of contrast was judged by the density level of the tumor and its vessel, evaluating the results in Hounsfield units (HU).

### 2.7. Animal Study

All experimental protocols were approved by the Institutional Animal Care and Use Ethic Committee of N.N. Blokhin National Medical Research Center of Oncology. In vivo experiments were conducted using 8-week-old outbred adult male rats (n = 6) with a body weight of 80–100 g (from Breeding Facility of N.N. Blokhin National Medical Research Center of Oncology, Moscow, Russia). PC-1 cells (obtained from the collection of Laboratory of biochemical basics of pharmacology and cancer models of N.N. Blokhin National Medical Research Center of Oncology, Moscow, Russia) were implanted intramuscularly [49].

In brief, the implantation of PC-1 cells was carried out in the posterior group of the femoral muscles (obtains blood supply from a. femoralis). The cells were implanted as 0.25 mL of a 20% suspension of tumor tissue homogenate from donor rat in Hanks solution. MNSs in the concentrations of 200 μg/mL (by IONPs) in sterile water for injections in single doses of 0.1 mL (20 μg), 0.2 mL (40 μg), or 0.3 mL (60 μg) per tumor were administered intraarterially after the tumor nodule reached the volume of 6.5 cm^3^. MNSs were stored no longer than three days after preparation before their administration to animals.

Surgical access to the femoral artery of rats was performed under general anesthesia via Zoetil-100 (Virbac, Carros, France). The injection was carried out by an infusion system composed of an intravenous peripheral catheter G27 (Troge, Hamburg, Germany) and a 2.0 mL plastic syringe. In vivo contrast assessment was performed over 30 min and 14 days after intraarterial administration.

The rats’ behavior, local effects, and body weight were monitored every second day for the three doses of the injected sample (20, 40, 60 μg IONPs per tumor). Local irritant effect, pain, tissue ischemia (cyanosis, edema, necrosis, tissue rejection), and self-amputation of the paw were also analyzed.

All animal experiments were conducted in accordance with the internationally accepted principles for laboratory animal use and care, as described in the EU Directive 2010/63/EU, and with approval from the Ethics Committee for Animal Research of the N.N. Blokhin Cancer Research Center.

### 2.8. Statistical Analysis

To evaluate the accuracy of the used experimental methods, all measurements including rat experiments were conducted for at least three parallel independent samples. The obtained data are presented as the means with standard deviation.

## 3. Results

### 3.1. Assessment of the Thickness and Stability of the HSA Coating by DLS

**Figure 2:**
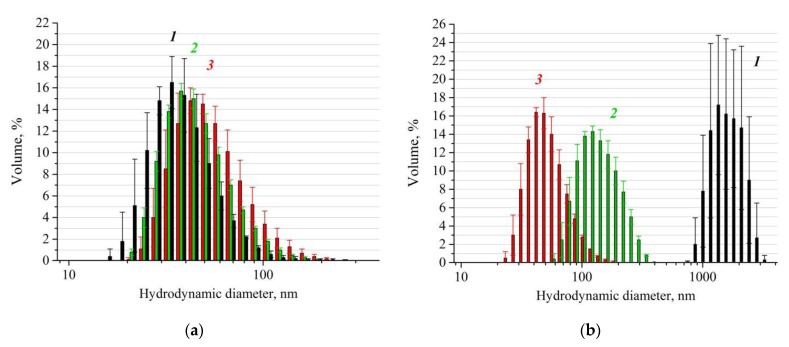
Dynamic light scattering (DLS) size distribution by volume for particles in the control sample “N0” (1, black) and the samples “NH0” (2, green) and “NH1” (3, red): (**a**) After overnight incubation; (**b**) after 30 min of incubation, dilution in 0.05 M phosphate buffer pH 6.3 followed by IgG addition and overnight incubation. C_IgG_/C_IONPs_ = 0.6 [mg/mg].

The average hydrodynamic diameters of the particles that have the maximal contribution to the volume distributions increased in the order “N0” < “NH0” < “NH1” (Figure 2; see Figure S2 for more details). The thicknesses of the protein coating on the surface of the nanoparticles could be estimated from the average hydrodynamic diameters of the particles based on the assumption of protein adsorption on individual nanoparticles. In our experiments, the thicknesses of the protein coating were ~ 4 ± 1 nm in the case of “NH0” and ~ 7 ± 1 nm in the case of “NH1”, which can be also seen in Figure 2a.

As above-mentioned in Section 2.2 and as expected from the previous data [36], the high affinity of an immunoglobulin G to the IONP surface must promote the substitution of HSA on the IONP surface with the formation of micron-sized aggregates in the case of the low stability of the HSA coating. An analysis of the particle sizes by DLS in the samples after the addition of an immunoglobulin G solution was performed. The addition of IgG led to the formation of aggregates with micron sizes in the absence of HSA in the samples, whereas in the presence of HSA, the particles sizes were below 1 µm and significantly depended on the presence of hydrogen peroxide in the samples: the aggregate sizes in “NH0” were larger than in “NH1” (Figure 2b). This means that the hydrogen peroxide promoted the enhanced immobilization of HSA on the surface due to oxidative modification of individual HSA molecules and/or the intermolecular cross-linking described in the papers [50,51,52,53,54]. It should also be emphasized that the efficiency of fixation of the HSA coating on the surface of IONPs was observed within 30 min after the beginning of the “NH1” incubation (for more details see Section S2 in Supplementary Materials). Based on the DLS confirmation of the HSA coating stability, the sample “NH1” was chosen for subsequent animal experiments. The average hydrodynamic diameters of the particles that had the maximal contribution to the volume and number distributions in “NH1” were d_V_ ~40 ± 1 nm and d_N_ ~35 ± 1 nm correspondingly. The volume and number distributions for MNSs administered to the rats’ tumors are given in the Supplementary Materials (Figure S3).

### 3.2. Evaluation of the Coating Stability by EMR Spectroscopy

The variation in the relative thickness of the coatings on IONPs could be estimated from the EMR spectra. The magnitude H_1_ of the local magnetic field induced by particles in the aggregates formed in an external magnetic field [45,55,56] is proportional to magnetic dipole moments µ_i_ induced by different particles and is inversely proportional to the cube of interparticle distance in the aggregates [57]. The local magnetic field causes a shift in the EMR spectrum that is proportional to H_1_ [58]. According to the resonance conditions, the spectrum of IONPs, which are involved to some degree in the formation of linear aggregates, is located (or shifted) in lower fields (in higher g-factors) relative to the spectrum of individual particles. Furthermore, the smaller the distance between the IONPs, the lower the magnetic field of the spectrum center position. This phenomenon was used for the layer thickness evaluation in our previous studies [30,36].

**Figure 3:**
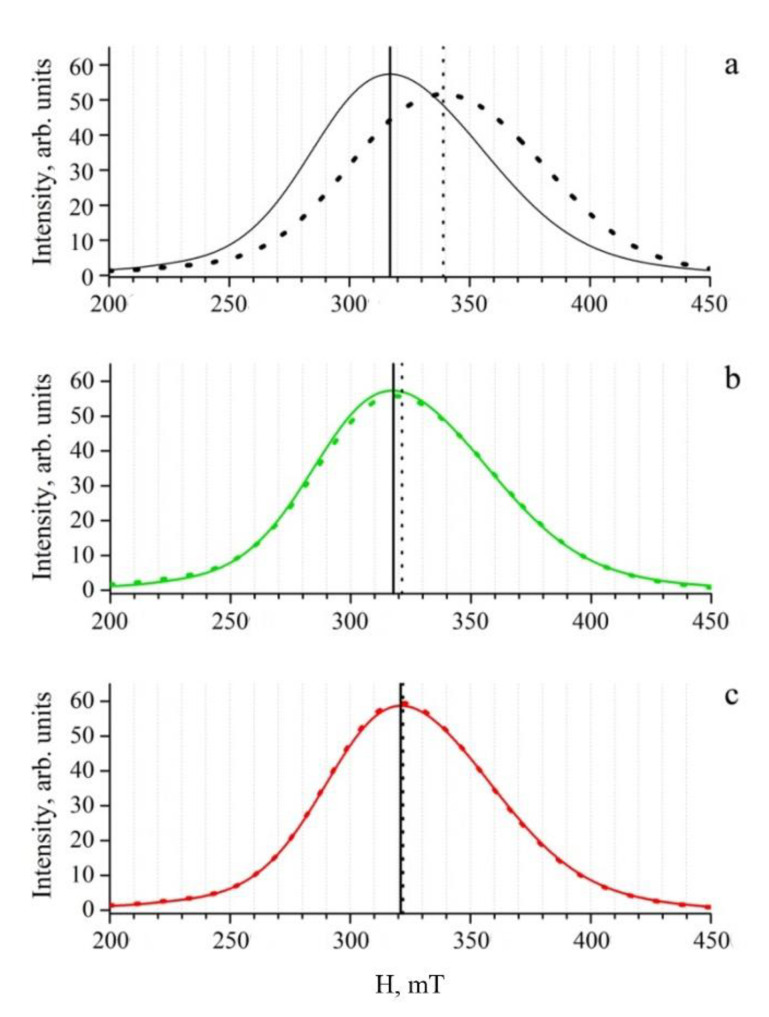
Electron magnetic resonance (EMR) absorption curves of the diluted samples “N0” (**a**), “NH0” (**b**), “NH1” (**c**) before (all solid lines) and after (all dotted lines) the addition of immunoglobulin G. Immunoglobulin G was added after the overnight incubation of samples. The IONP concentration in all of the analyzed samples was 0.05 mg/mL. The resonance field of the corresponding spectra before and after the addition of IgG are marked in the figure.

**Figure 4:**
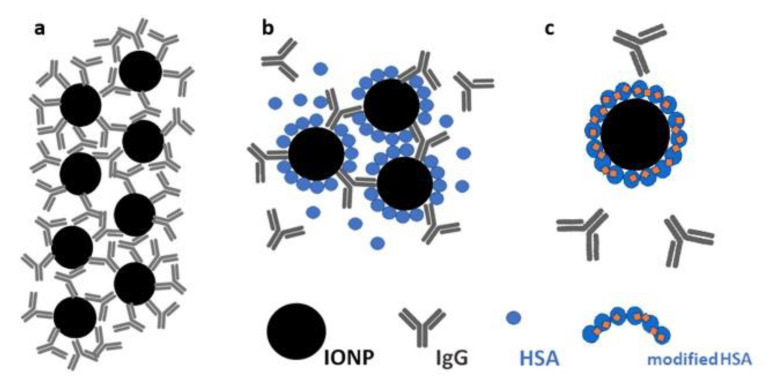
Schematic presentation of the interaction between IONPs and protein in the samples of “N0” (**a**), “NH0” (**b**), and “NH1” (**c**) after the addition of IgG to the solutions.

Figure 3 shows the absorption curves obtained by the means of the integration of the experimental EMR spectra of the samples “NH1”, “NH0”, and “N0” before and after interaction with IgG. The maximum of the absorption curve corresponds to the resonance field (the position of the spectrum center, g-factor of EMR signal). As demonstrated in Table 1, for the control sample “N0”, the value of the g-factor before the addition of IgG was higher compared to the samples containing HSA. This means that the HSA in the solution interacts with magnetic nanoparticles, preventing their aggregation in an external magnetic field. The addition of immunoglobulin G in IONP solutions changed the g-factor values in all of the studied samples. The greatest shift in the g-factor to higher values was observed for the control sample. This corresponded to the formation of micron-sized aggregates detected by DLS. Possible aggregates raised in the solution, in this case, are schematically presented in Figure 4a.

As has already been mentioned (see Section 2.2 and Section 3.1), due to the high affinity to the IONP surface, immunoglobulin G can replace some unfixed HSA on the surface. In our opinion, such a process took place in the presence of IgG in the solution of the sample “NH0” (Figure 4b). Nevertheless, by the differences between g-factors, the non-stability of the HSA coating in “NH0” could be detected during the magnetic separation (see Section S3 in the Supplementary Materials).

An insignificant g-factor shift in the EMR signal was measured for the sample “NH1” after the addition of IgG in the solution. In our opinion, this is due to the almost unchangeable local environment of magnetic nanoparticles consisting of fixed HSA molecules. Immunoglobulin G did not substitute the FRA-modified HSA molecules on the IONP surface in sample “NH1” (Figure 4c).

The g-factor shift of the EMR signal as a result as IgG addition in the solution decreased for the samples in the order: “N0” > “NH0” > “NH1”. The results obtained on the substitution of unfixed HSA on the IONP surface in the sample “NH0” by IgG and the retention of HSA coatings in the sample “NH1” were consistent with the data obtained using the DLS method and described above. The data on the samples with different incubation duration (Supplementary Materials Table S1) underline that the HSA coating stability depends on the incubation duration and the presence oof hydrogen peroxide. Thus, both the DLS and EMR methods allowed us to conclude that the HSA coating stability on the surface of the IONPs was increased as a result of the addition of hydrogen peroxide.

The amount of protein attached on the surface of the IONPs was studied using the Bradford protein assay. The results are presented in Supplementary Materials Section S4.

### 3.3. Characterization of MNSs before In Vivo Administration

Based on the data of the HSA coating stability obtained by the DLS and EMR methods as well as the data on the amount of the protein in the adsorption layer, we selected sample “NH1” for the injection to rat tumors (inoculated hepatocellular carcinoma PC-1), following the computed tomography analysis. The sample “NH1” was characterized by d_N_ ~35 nm by DLS (see Supplementary Materials Section S2 and Figure S3 for details), consisted of IONPs with the stable HSA coating, and was treated by magnetic separation to remove the excess components. The concentration of the MNS hydrosol taken for the analysis corresponded to the IONP concentration of 200 μg/mL and was evaluated by EMR using the IONPs studied before as the reference [35].

As has been stated above, the MNSs were stored for no longer than three days before administration to the animals. The peroxidase-like activities of IONPs in MNSs (sample “NH1”), “NH0”, and “N0” in the reaction of OPD oxidation was evaluated by V_max_ on the first day after the magnetic separation and decreased in the order: “N0” > “NH0” > “NH1”. The colorimetric test also showed some decrease in the V_max_ during subsequent storage of the MNSs for seven days. The value of the relative speed V_max(MNSs)_/V_max(“N0”)_ (%), which was ~30 ± 3% on the first day, decreased to ~16 ± 2% on the seventh after MNS preparation.

Before the injection was given to the rats, the MNS hydrosol was put in a syringe to test the detectability of the MNSs by computed tomography (Figure S5). The MNSs were shown to have a pronounced contrast level of −54 to −120 HU units, which corresponds to the density of adipose/connective tissue.

### 3.4. In Vivo Detection of MNSs by the Computed Tomography

**Figure 5:**
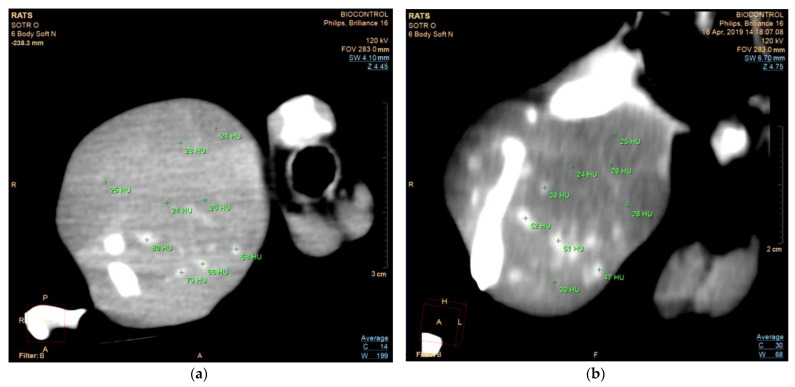
The cross-sectional images of the tumor nodule in the rat femoral muscle from two different angles after 14 days of the intraarterial administration of “NH1”: (**a**) transverse and (**b**) sagittal planes. Additional cross-sectional images of the tumor nodule are presented in Supplementary Materials Section S5.

The results of the in vivo CT study of the tumor nodule using three doses of the injected sample “NH1” (20, 40, 60 μg IONPs per tumor) indicated accumulation of MNSs in the tumor blood vessels. The cross-sectional images of the tumor nodule in the rat femoral muscle after the MNS injections are shown in Figure 5 and in Supplementary Figures S6–S8. The changes in the X-ray attenuation values after MNS injection are marked in these figures.

First of all, we note that the contrast by MNSs was equivalent to different doses of the MNSs administered (Figures S7 and S8). As a result of the injections for some of the tumor vessels, the vascular density ranged from −47 to −80 HU while the vessels without MNSs were not detectable on the tissue background (the tumor tissue density ranged from −24 to −26 HU before and after the MNS injections).

Furthermore, it is worth noting here that the contrast of vessels revealed 30 min after their intraarterial administration remained during the observation period of 14 days. The CT measurements showed a non-dose-dependent contrast of the tumor vascular bed. We suppose that this could be explained by limitations in the blood volume in the tumor. Therefore, the minimal dose (20 μg IONPs per tumor) seems to be enough for the noticeable contrasting of tumor vessels.

The administration of MNSs (“NH1”) to rats in all of the studied doses (20, 40, 60 μg IONPs per tumor) did not lead to immediate or delayed adverse reactions. No significant systemic toxic effects were recorded during the 14 days.

## 4. Discussion

The albumin-modified IONPs are well-known nanosystems for drug transport [59], imaging [60,61], and for cancer treatment with photodynamic therapy [62]. Recently, it has been shown that albumin-modified IONPs can be used for prolonged drug release at the desired location in a rat model of retinal degeneration: the IONPs were detected by MRI up to 30 weeks in the injection area without changes in the retinal structure and function during suprachoroidal delivery [63]. The authors of [64], who detected the signal of Fe_3_O_4_/TaO_x_ core/shell nanoparticles in blood vessels for more than 2 h, underlined that imaging of the blood vessels using CT is advantageous for cancer therapy because the tumor-associated vessel is one of the main targets in effective cancer treatment. Prominent accumulation and long-term retention (more than 7 days in the tumor tissue) of nanoparticles with a HSA coating are underlined to be important for imaging and phototherapy [65,66].

Taking into account a large number of studies devoted to efficient drug transport by HSA on the surface of nanoparticles, HSA functionalization, HSA property to enhance the circulation of particles in the bloodstream, and hyperthermia with the aid of IONPs, we suppose that the therapy of tumors and multimodal imaging [11,13] (i.e., simultaneous diagnostics by CT and MRI) by our MNSs could also be carried out. Since the administration of MNSs (“NH1”) to rats did not lead to immediate or delayed adverse reactions and no significant systemic toxic effects were recorded during the observation period of 14 days, we hypothesize that the two week long influence on the tumor growth should be studied in the future. No doubt that all potential modalities of MNS application require additional study of the retention time of the IONPs in tumors and other organs that can depend or not depend on the method of the particle administration [67].

According to our experimental data (the stability test based on IgG interaction with IONPs), the protein coating obtained under conditions of free radical generation on the IONP surface was more stable than the protein coating we obtained by the physical adsorption of HSA on the surface of the IONPs. These results led us to expect that the protein coating stability in physiological conditions would also take place.

Since the IONPs under study had peroxidase-like activity, and the detected •OH generation in the IONP hydrosol decreased in the presence of HSA according to [46], we carried out a colorimetric test to analyze the changes in hydroxyl radical generation by the peroxidase-like IONPs due to HSA fixation on their surface by adsorption and FRA. According to the data obtained, peroxidase-like activity decreased in the sequence of the samples: “N0” > “NH0” > “NH1”. These data allow us to suppose that MNSs could be characterized by lower toxicity in vivo than bare IONPs or IONPs with physically adsorbed HSA. While explaining these results, it should be taken into consideration that (1) the treatment of IONPs with the hydrogen peroxide in FRA could reduce the ability of IONPs to participate in the generation of radicals due to the conversion of ferrous ions on the surface of the IONPs to ferric, and/or (2) HSA coating on the surface of the IONPs in “NH1” is denser compared to “NH0”. Therefore, since MNSs have residual peroxidase-like activity for at least seven days after their synthesis, MNSs could be expected to have the ability to catalyze the generation of reactive oxygen species (ROS) in vivo, induce cell damage, and be effective for the suppression of tumor growth by ferroptosis [15].

Thus, the obtained stable fixation of the MNSs in small vessels of the tumor and the successful imaging of the bed of the tumor vessels by CT suggest that the designed MNSs (IONPs coated with HSA by FRA) have the potential for use in long-term tumor treatment including therapy and diagnostics, particularly in epidermal diseases [68] and for phototheranostic applications in the treatment of deeply seated tumors [69]. It is worth noting here that our experiments do not provide answers to all of the questions that have arisen regarding the in vivo behavior of the HSA-coated IONPs and the toxicity effects expected after a longer exposure of MNSs in the tumor. Detailed toxicological studies as well as longer detection of IONPs in tumor tissue will be taken into consideration in the next steps of our research.

## 5. Conclusions

Nowadays, the applications of IONPs as a core and HSA as a coating, or as a dominant component of particles, are very popular. There are some commercial, particularly, FDA approved, examples of both types: those based on IONPs (i.e., Feraheme (ferumoxytol), Nanotherm [70]) and those consisting of HSA (i.e., Optison, Albunex [71]).

In the study, magnetic nanosystems (MNSs) based on iron oxide nanoparticles coated with human serum albumin immobilized on their surface were synthesized using an original free radical approach (FRA) and for the first time administered to tumors. It has been proven by DLS and EMR spectroscopy that FRA allows obtaining a stable coating on IONPs with peroxidase-like activity. The estimated thickness of the protein coating on the surface of IONPs was ~7 ± 1 nm. To confirm the fixation of HSA on the surface of the IONPs, an original test based on the affinity of immunoglobulin G to IONPs was used. The peroxidase-like activity of MNSs has been proven to be lower than the activity of bare IONPs and IONPs coated by physically adsorbed HSA.

Computed tomography (CT) analysis was performed after the synthesized MNSs were injected into rats with an inoculated hepatocellular carcinoma PC-1. The concentration of MNS hydrosol taken for the CT analysis corresponded to a IONP concentration from 20 to 60 µg per tumor. The CT carried out 30 min and 14 days after their intraarterial administration showed a non-dose- and non-time-dependent contrasting of the tumor vascular bed. Therefore, the minimal dose (20 μg IONPs per tumor) seems to be sufficient for noticeable contrasting of the tumor vessels. To the best of our knowledge, the data on the CT contrast obtained with the aid of IONPs modified with HSA are published for the first time. Furthermore, albumin-functionalized IONPs obtained by means of free radical modification of the HSA molecules on the surface of the IONPs were administered to animals for the first time in the study.

We hope that our particles, characterized by peroxidase-like properties and a stable albumin coating obtained without toxic cross-linkers, demonstrating the long-term presence in tumor, are suitable for in vivo multimodal imaging and theranostics. A detailed study of the properties of MNSs is needed.

## Figures and Tables

**Table 1:** Experimental g-factors of the EMR signals of IONPs in samples with and without immunoglobulin G (IgG).

Sample	without IgG	with IgG
“N0”	2.222 ± 0.006	2.080 ± 0.006
“NH0”	2.218 ± 0.006	2.195 ± 0.006
“NH1”	2.198 ± 0.006	2.196 ± 0.006

## Data Availability

Data is contained within the article, the Supplementary Material and available on request from the corresponding author.

## Supplementary Materials

The following supporting information can be downloaded at: https://www.mdpi.com/article/10.3390/pharmaceutics14122771/s1—A file divided into several sections (S1, S2, S3, S4, S5) containing text, one table, and several figures. Section S1: “Nanoparticle Tracking Analysis (NTA) data” contains Figure S1. NTA particle size distribution of the MNP hydrosol. Section S2: “Dynamic light scattering (DLS) data” contains text and two figures. Figure S2. Autocorrelation curves (autocorrelation coefficient versus time curves) of DLS corresponding to the control sample “N0” (1, black) and the samples “NH0” (2, green) and “NH1” (3, red): (a) After overnight incubation; (b) after 30 min of incubation, dilution in 0.05 M phosphate buffer pH 6.3 followed by IgG addition and overnight incubation. Figure S3. DLS size distribution by volume (a), number (b), and intensity (c) for particles in the sample “NHInfluence of stabilizing components on 1”. Section S3: “Electron magnetic resonance (EMR) data” contains text, Table S1. Experimental g-factors of EMR signals of the IONPs in samples containing immunoglobulin G (IgG). IgG was added to the samples with different incubation times. Figure S4. EMR absorption curves of the diluted samples “NH1” (a) and “NH0” (b) before (red and green lines) and after (wine and olive lines) magnetic separation. The magnetic separation was carried out after overnight incubation of the samples. The IONP concentration in all of the analyzed samples was 0.05 mg/mL. The g-factors of the resonance field of the corresponding spectra are marked in the figure. Section S4: “The estimation of the amount of protein on the surface of nanoparticles” contains text. Section S5: “MNSs detection by the computed tomography” contains figures. Figure S5. The MNS hydrosol detection by computed tomography in a 0.5 mL insulin syringe. IONP concentration in the hydrosol equals 200 μg/mL. Figure S6. The cross-sectional computed tomography images (unmarked (upper) and marked (bottom)) of the tumor node from two different angles (transverse and sagittal planes) in the rat femoral muscle after 30 min of MNS (“NH1”) intraarterial administration to the rats with hepatocarcinoma HCC transplanted intramuscularly. The amount of MNSs injected corresponded to the IONP amount of 60 μg; Figure S7. The cross-sectional computed tomography images (unmarked (upper) and marked (bottom)) of the tumor node from two different angles (transverse and sagittal planes) in the rat femoral muscle after 14 days of MNS (“NH1”) intraarterial administration to rats with hepatocarcinoma HCC transplanted intramuscularly. The amount of MNSs injected corresponded to the IONP amount of 20 μg; Figure S8. The cross-sectional computed tomography images (unmarked (upper) and marked (bottom)) of the tumor node from two different angles (transverse and sagittal planes) in the rat femoral muscle after 14 days of MNS (“NH1”) intraarterial administration to rats with hepatocarcinoma HCC transplanted intramuscularly. The amount of MNSs injected corresponded to the IONP amount of 60 μg.
