# Supplementary material for: Albumin-Functionalized Iron Oxide Nanoparticles for Theranostics: Engineering and Long-Term In Situ Imaging
Source: Pharmaceutics. 2022 Dec 11;14(12):2771. doi: 10.3390/pharmaceutics14122771 (PMC9782891; doi:10.3390/pharmaceutics14122771)
Supplement: Supplementary file 1 [file pharmaceutics-14-02771-s001.zip › pharmaceutics-2040860-supplementary.pdf]

S1. Nanoparticle Tracking Analysis (NTA) data

Number-weighted particle size distribution and total particle concentration of the initial IONP hydrosol obtained after removing some largest particles described in Section 2.1 were measured with Nanoparticle Tracking Analysis (NTA) technique using the Nanosight LM10-HS instrument (Nanosight Ltd., UK) equipped with a 405 nm, 65 mW laser and a high-sensitivity Andor Luca (Andor Technology Ltd, UK) camera of EMCCD type. All measurements were performed according to the ASTM E2834-12(2018) standard [38].

For NTA, IONP hydrosols were diluted in deionized water down to concentration around 1.0-1.5×10^8 particles per ml, which is optimal for NTA. 18 videos of Brownian motion were recorded under following camera setups: Camera Shutter = 1000, Camera Gain = 450, Lower Threshold = 910, Higher Threshold = 9295, Duration = 60 s. Videos were processed in NTA 2.3 build 33 software (Nanosight Ltd., UK) using following setups: Detection Threshold = 9 multi, MinExpectedParticleSize = 30 nm. Tracks from all videos (total 8800) were collected together to obtain the number-weighted particle size distribution (PSD) and total particle concentration, corrected for dilution factor. Resulting PSD for sample "N0" is shown in Figure S1.

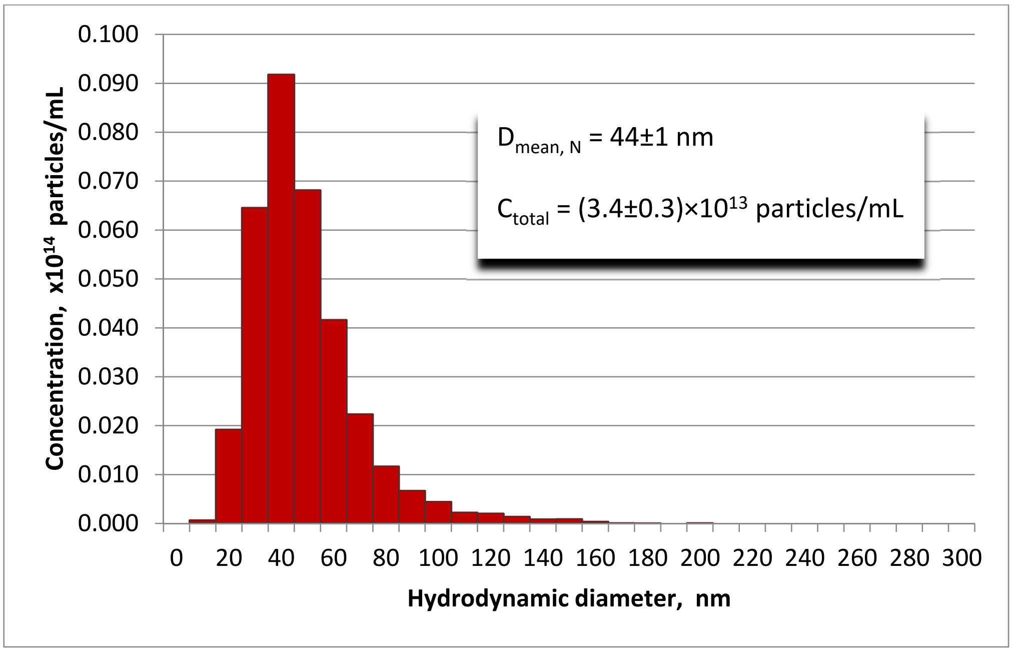

Figure S1. NTA particle size distribution of the MNP hydrosol.

Although NTA measurement results in PSD, evaluation of the exact total surface area of particles is not possible, as it has been shown in [39] due to distribution broadening caused by finite track length effect. Instead, from the NTA data we could evaluate the lower and upper boundaries for total surface area, using the relation d_N(NTA) ≤ d_S < d_S(NTA), where d_N(NTA) is a NTA mean number-weighted particle size; d_S is a real mean surface-weighted particle size; d_S(NTA) is a mean surface-weighted particles size, derived from measured NTA PSD. For IONP hydrosol under study d_N(NTA) was measured to be 44±1 nm, d_S(NTA) was equal to 66±3 nm. Thus, the total surface area of IONPs in the hydrosol (the sample "N0") were between 2.1×10^{17} and 4.6×10^{17} nm^2/mL.

[38] ASTM E2834-12(2018), Standard Guide for Measurement of Particle Size Distribution of Nanomaterials in Suspension by Nanoparticle Tracking Analysis (NTA), ASTM International, West Conshohocken, PA, www.astm.org, (2018). https://doi.org/10.1520/E2834-12R18.

[39] D. Tretiakova, N. Onishchenko, I. Boldyrev, I. Mikhalyov, A. Tuzikov, N. Bovin, E. Evtushenko, E. Vodovozova, Influence of stabilizing components on the integrity of antitumor liposomes loaded with lipophilic prodrug in the bilayer, Colloids Surfaces B Biointerfaces. 166 (2018) 45–53. https://doi.org/10.1016/j.colsurfb.2018.02.061.

S2. Dynamic light scattering (DLS) data

DLS volume distributions of particles sizes in the control sample “N0” and the samples “NH0” and “NH1” are presented in Figure 2. According to these data, particles of control sample “N0” are slightly less in size than in other two samples. The autocorrelation curves for these samples are shown in Figure S2a. Overlapping of the curves with each other suggests similarity in the particle sizes. Obtained DLS data (Figure 2b) on all samples indicate the change in particle diameters after interaction with IgG. It was found that particles of the sample “N0” have maximum size (diameter) after interaction with IgG. All the autocorrelation curves (Figure S2b) show almost similar exponential decay, suggesting the measured particles are mostly monodisperse in nature.

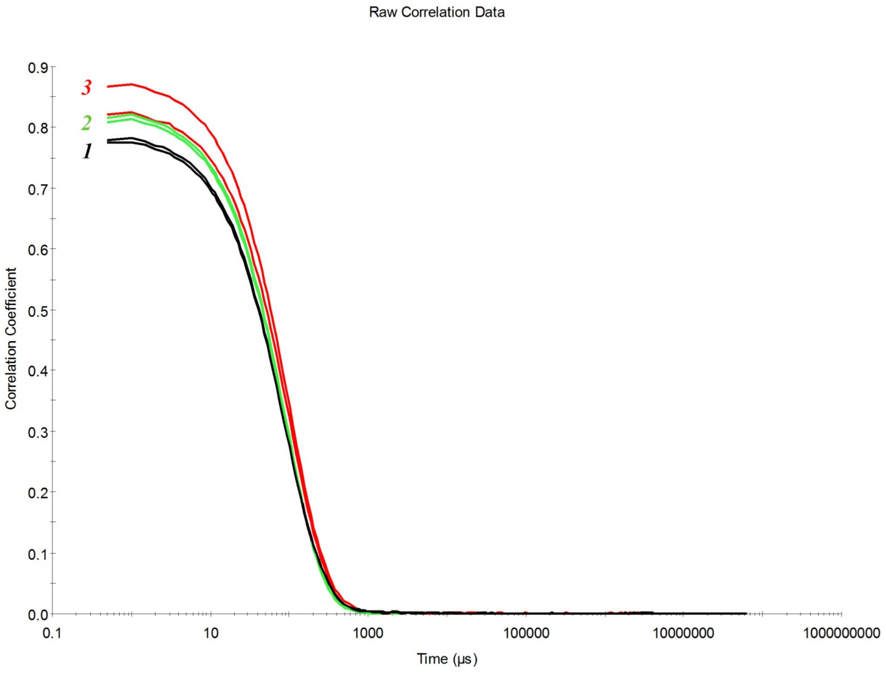

(a)

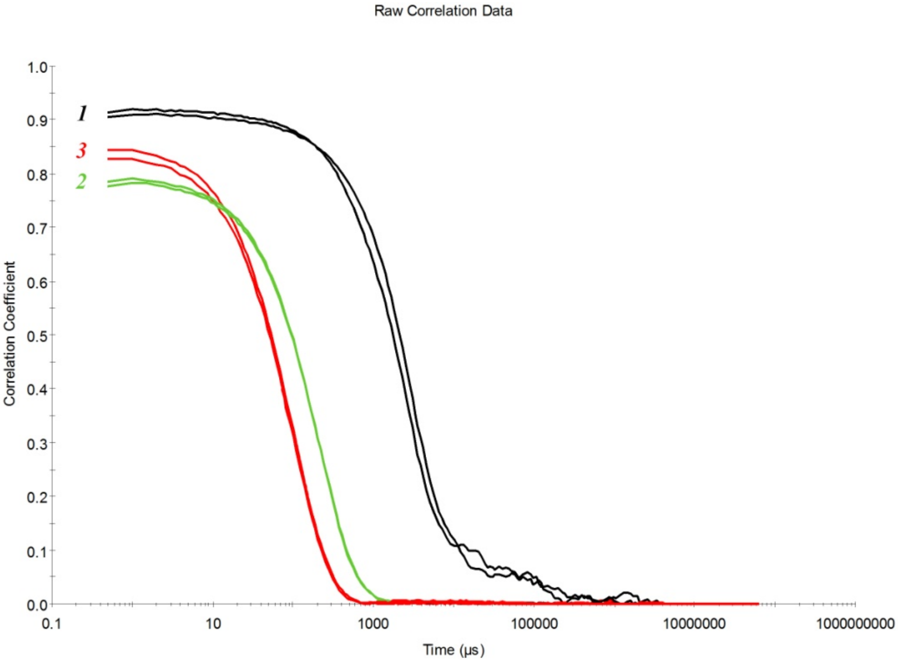

(b)

Figure S2. Autocorrelation curves (autocorrelation coefficient versus time curves) of DLS corresponding to the control sample “N0” (1, black) and the samples “NH0” (2, green) and “NH1” (3, red): (a) – after overnight incubation; (b) – after 30 minutes of incubation, dilution in 0.05M phosphate buffer pH 6.3 followed by IgG addition and overnight incubation.

Both in the case of 30-minute incubation and in the case of overnight incubation, the Z-average sizes of the aggregates in “NH0” exceeded 100 nm, while the particle diameters in “NH1” did not exceed 68 nm, remaining close to the particle sizes before IgG addition.

The volume and number distributions for magnetic nanosystems (MNSs) administered to the rats tumors were obtained after magnetic treatment of the sample “NH1” for removing the protein excess. The average hydrodynamic diameter that has the maximal contribution to the volume distribution is dv ~ 40 ± 1 nm (Figure S3a). The average hydrodynamic diameter that has the maximal contribution to the number distribution is dv ~ 35 ± 1 nm (Figure S3b). The intensity distribution for the sample “NH1” is presented in Figure S3c.

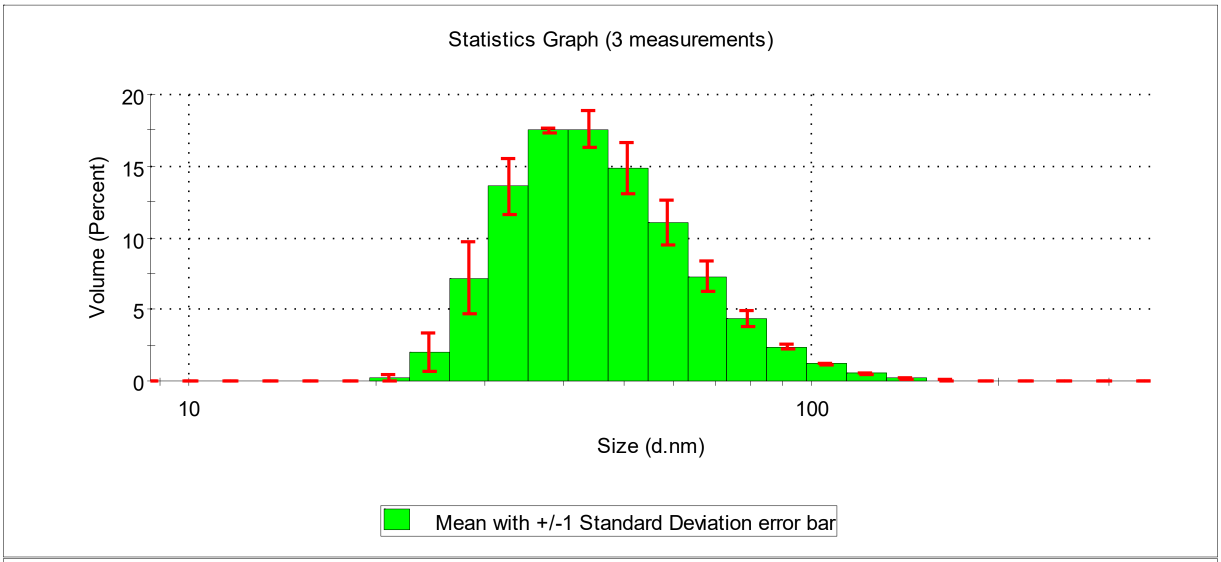

(a)

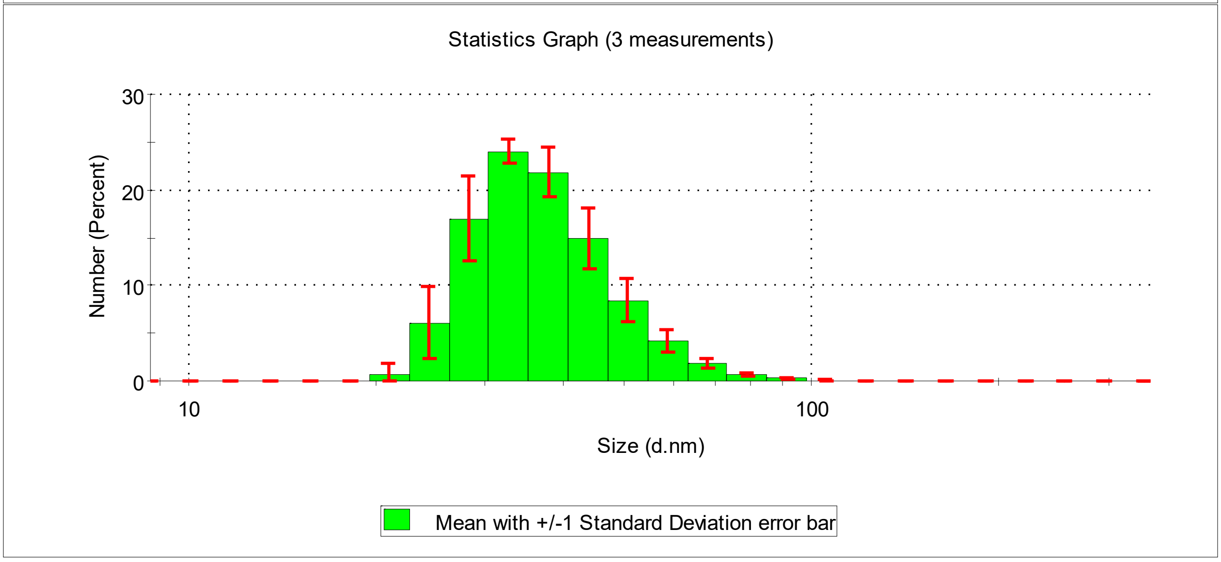

(b)

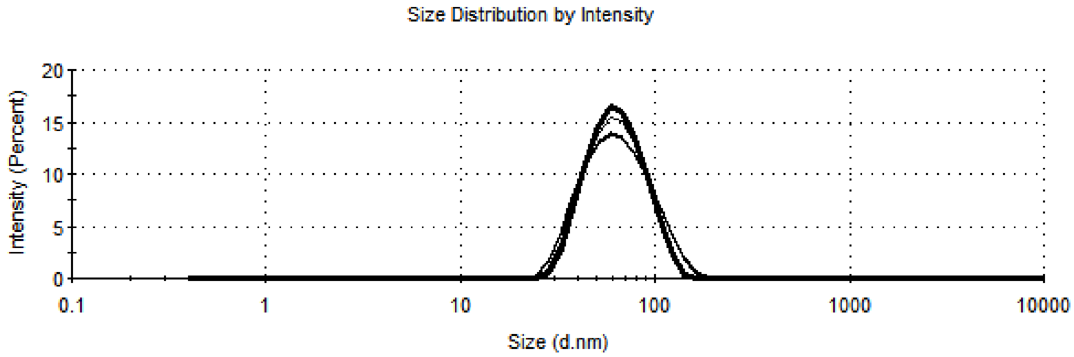

(c)

Figure S3. DLS size distribution by volume (a), number (b) and intensity (c) for particles in the sample “NH1”.

S3. Electron magnetic resonance (EMR) data

Immunoglobulin G was added after 15 minutes, 30 minutes, 60 minutes and overnight incubation of samples “NH0” and “NH1”. It could be indicated that the g-factors for both the samples (Table S1) become higher with increase of the incubation duration. This fact as well as the peculiarities of IgG interaction with the samples (Figures 2, 3) further confirms the formation of adsorption layer of HSA on the surface of the IONPs that disturbs the interaction between magnetic nanoparticles.

Table S1. Experimental g-factors of EMR signals of the IONPs in samples containing immunoglobulin G (IgG). IgG was added to the samples with different incubation times.

Incubation duration	“NH1”	“NH0”
15 min	2.189±0.006	2.178±0.006
30 min	2.194±0.006	2.192±0.006
60 min	2.199±0.006	2.197±0.006
overnight	2.198±0.006	2.195±0.006

The comparison of the absorption curves for the samples “NH1” and “NH0” before and after magnetic separation has also shown a slight change of the “NH0” spectrum (Figure S4). The lower fields (and higher g-factors) confirm some HSA loss in “NH0” due to the process of removing protein excess. On the contrary, for “NH1” a significant amount of HSA seems to retain on the surface of the IONPs after magnetic separation. Therefore, the bonding of free radically oxidized albumin on the surface of the particles could be expected in “NH1” and the HSA coating on IONPs could be considered rather stable to be used for administration to animals.

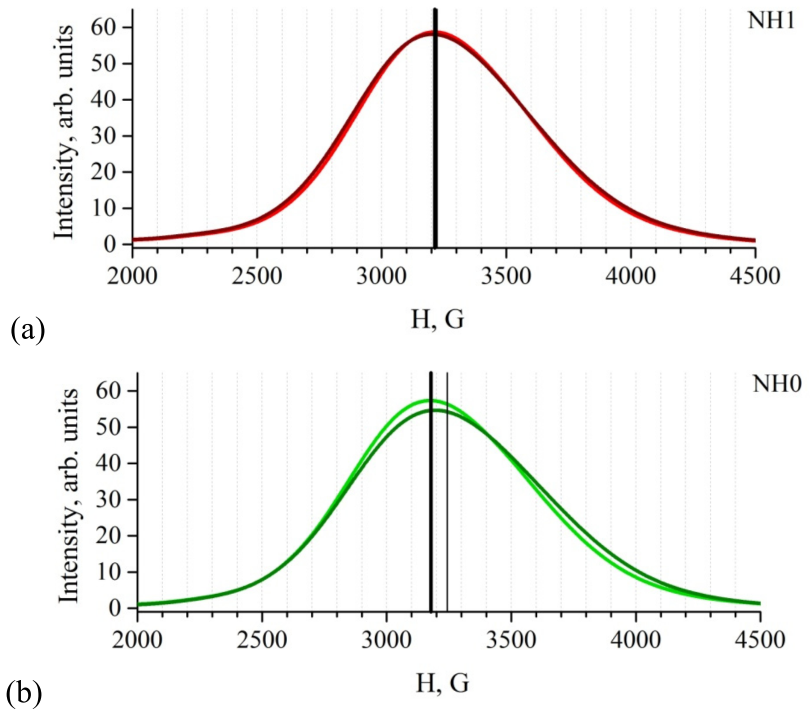

Figure S4. EMR absorption curves of the diluted samples “NH1” (a) and “NH0” (b) before (red and green lines) and after (wine and olive lines) magnetic separation. The magnetic separation was carried out after overnight incubation of the samples. The IONP concentration in all of the analyzed samples was 0.05 mg/mL. The g-factors of the resonance field of the corresponding spectra are marked in the figure.

IgG addition induces the small g-factor change in EMR signal (about 0.023 g-factor units) for the sample before magnetic separation. This change is higher (about 0.110 g-factor units) for the sample incubated overnight after magnetic separation. The mentioned differences between g-factors could be explained by the non-stability of HSA coating in “NH0” that loses protein in solution during the magnetic separation.

S4. The estimation of the amount of protein on the surface of nanoparticles

The amount of HSA in the samples containing IONPs was estimated using the Bradford protein assay. The estimation was carried out at the three stages of magnetic separation in the supernatants to prove the protein excess removing (according to the description given in Section 2.1). The estimation was carried out in the samples in the presence and absence of hydrogen peroxide in the reaction systems. The solutions of HSA with the concentration as in the initial samples “NH1” and “NH0” were used as the control samples.

According to obtained results, the protein content in the “NH0” sample is at least ~ 0.26 mg protein per 1 mg of magnetic nanoparticles, and in the “NH1” sample is at least ~ 0.36 mg protein per 1 mg of nanoparticles. These data indicate that the presence of the hydrogen peroxide affects the amount of protein on the surface of the IONPs The amount is obviously associated with free radical modification of the protein leading to its enhanced bonding to the IONPs that corresponds to the data provided by DLS and EMR. As we can see some amount of protein in “NH0” sample is lost from IONPs surface at the magnetic separation process.

The number of protein molecules on the surface of each IONP in the “NH1” sample estimated from the NTA data (see Section A above in Supporting Materials) is at least ~ 100 molecules.

It should be noted that some protein amount could become undetectable for the Bradford protein assay. The protein detectability is directly associated with the protein binding to the nanoparticles surface, which makes it less accessible for Bradford's reagent, as well as with the influence of the scattering IONPs on the optical density at a wavelength of 595 nm, which we use for the quantitative determination of the protein in the Bradford protein assay. Consequently, using Bradford protein assay one could obtain only the relative but not the absolute amounts of protein.

S5. MNSs detection by the computed tomography

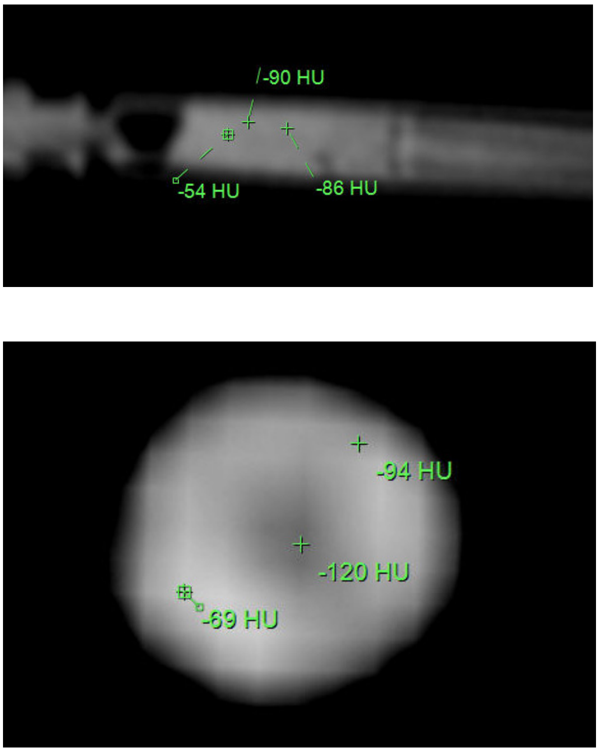

Figure S5. The MNS hydrosol detection by computed tomography in a 0.5 mL insulin syringe. IONP concentration in the hydrosol equals 200 μg/mL.

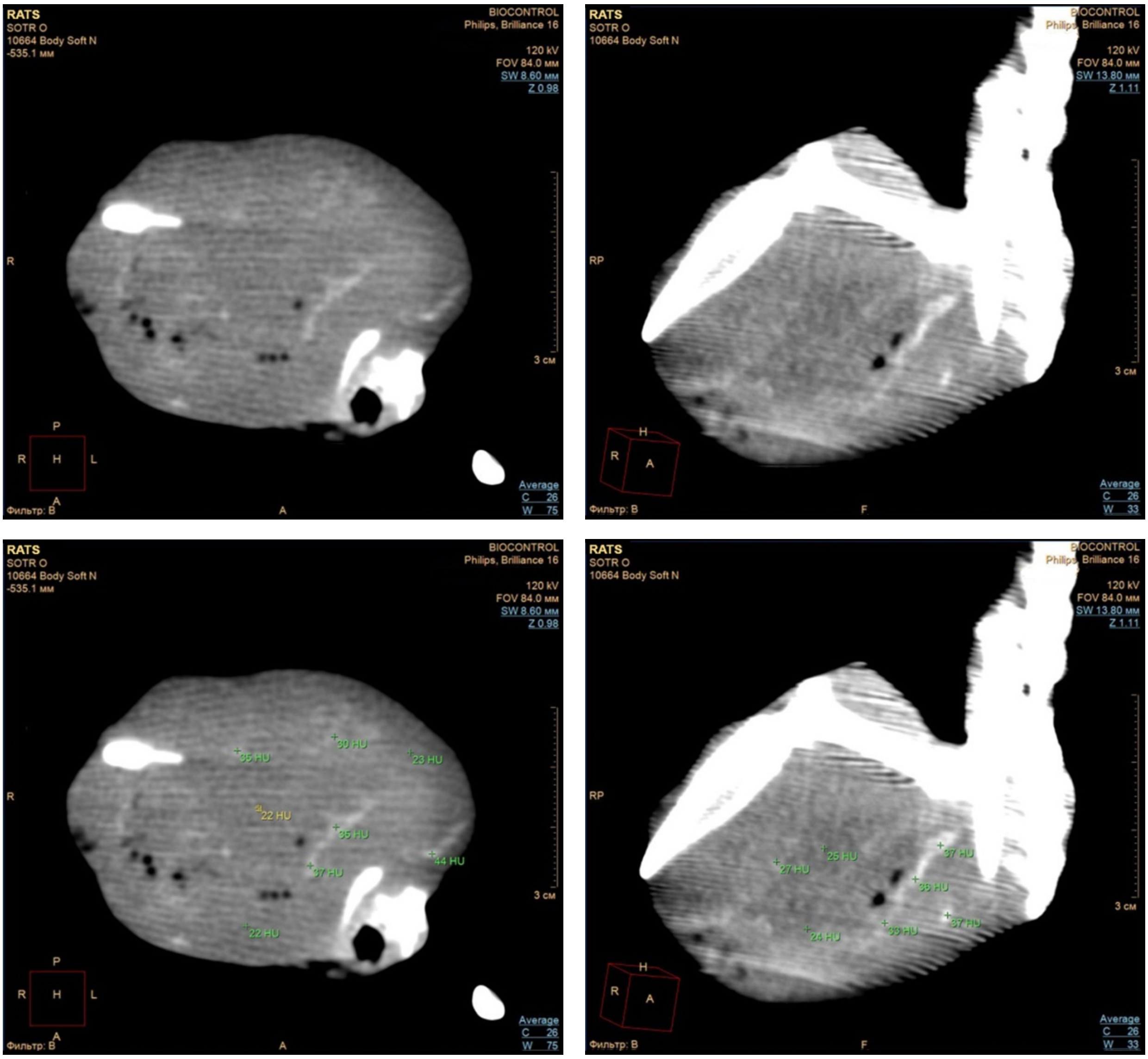

Figure S6. The cross-sectional computed tomography images (unmarked (upper) and marked (bottom)) of the tumor node from two different angles (transverse and saggital planes) in the rat femoral muscle after 30 minutes of MNS ("NH1") intraarterial administration to the rats with hepatocarcinoma HCC transplanted intramuscularly. The amount of MNSs injected corresponded to the IONP amount of 60 μg.

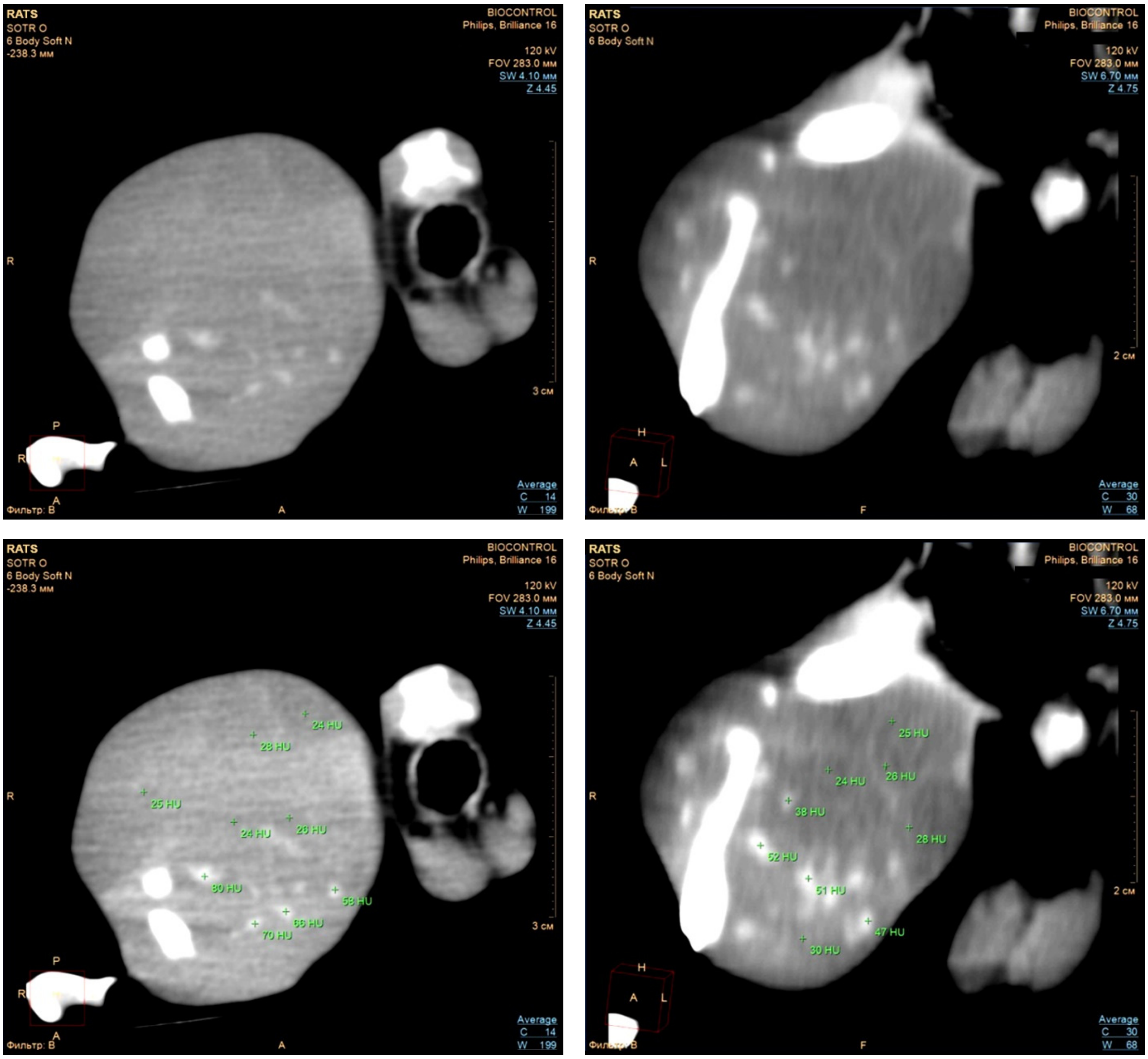

Figure S7. The cross-sectional computed tomography images (unmarked (upper) and marked (bottom)) of the tumor node from two different angles (transverse and saggital planes) in the rat femoral muscle after 14 days of MNS ("NH1") intraarterial administration to the rats with hepatocarcinoma HCC transplanted intramuscularly. The amount of MNSs injected corresponded to the IONP amount of 20 μg.

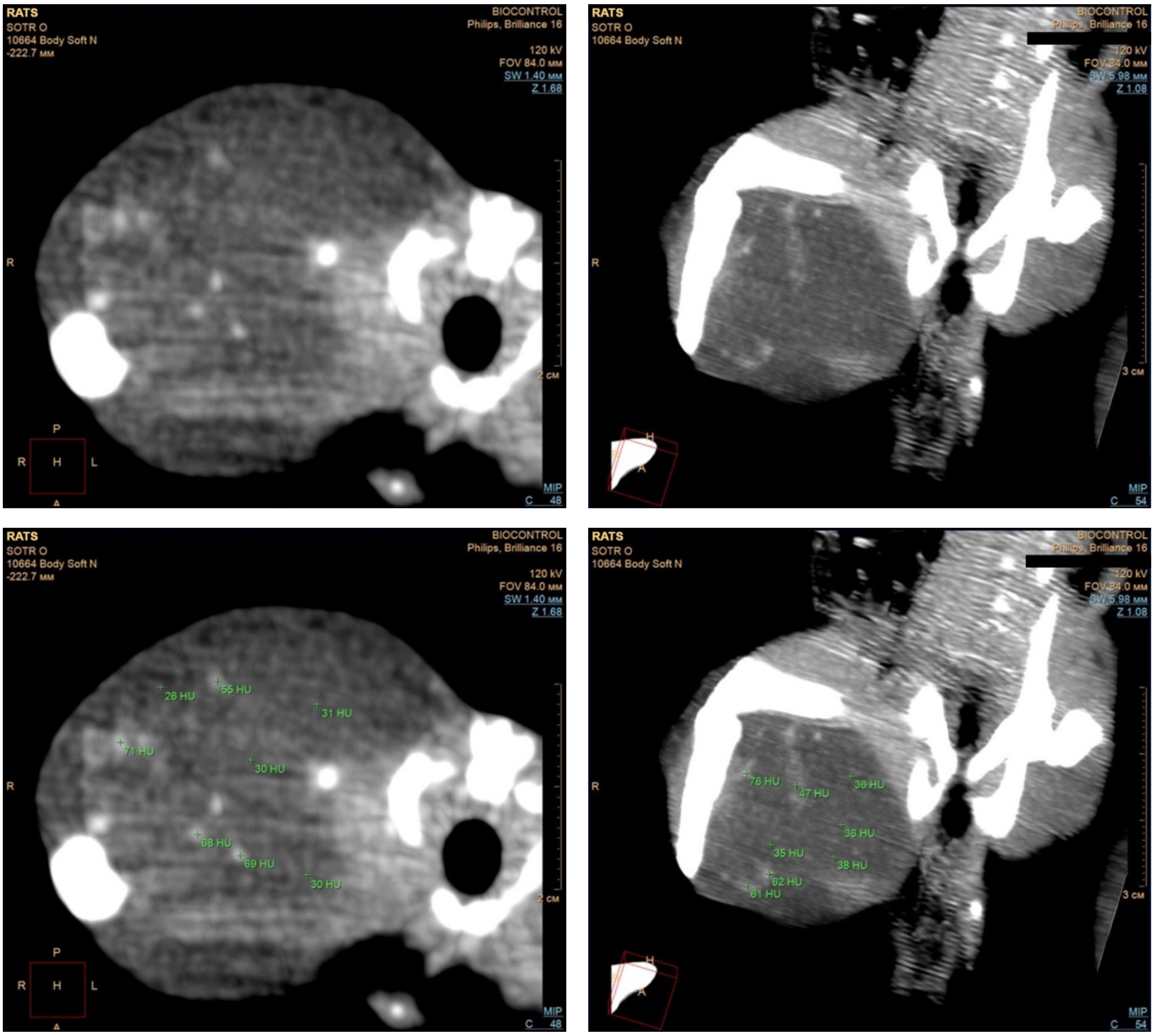

Figure S8. The cross-sectional computed tomography images (unmarked (upper) and marked (bottom)) of the tumor node from two different angles (transverse and saggital planes) in the rat femoral muscle after 14 days of MNS ("NH1") intraarterial administration to the rats with hepatocarcinoma HCC transplanted intramuscularly. The amount of MNSs injected corresponded to the IONP amount of 60 μg.
